# Hangover headache and its behavioral changes in rats

**DOI:** 10.22038/IJBMS.2023.66724.14644

**Published:** 2023-03

**Authors:** Shiguang Lu, Ying Zhang, Yuejun Yang, Yafang Zhang, Guangcheng Qin, Qingqing Fu, Yingying Shi, Fan Zhang, Zhe Wang, Yanhe Chen, Yuancai Liu, Lixue Chen

**Affiliations:** 1 Hubei Provincial Key Lab for Quality and Safety of Traditional Chinese Medicine Health Food, Hubei, China; 2 Laboratory Research Center, The First Affiliated Hospital of Chongqing Medical University, Chongqing, China

**Keywords:** Alcohol, Animal, Behavior, Central nervous system – sensitization, Disease models, Hangover, Headche

## Abstract

**Objective(s):**

The present study aims to establish and evaluate a rat model for hangover headaches caused by alcoholic drinks.

**Materials and Methods:**

Chronic migraine (CM) model rats were divided into 3 groups, and intragastrically administered alcoholic drinks (sample A, B, or C) to simulate hangover headache attacks. The withdrawal threshold for the hind paw/face and the thermal latency of hind paw withdrawal were detected after 24 hr. Serum was collected from the periorbital venous plexus of rats in each group, and enzymatic immunoassays were used to determine the serum levels of calcitonin gene-related peptide (CGRP), substance P (SP), and nitric oxide (NO).

**Results:**

Compared with the control group, the mechanical hind paw pain threshold was significantly lower in rats administered Samples A and B after 24 hr; however, no significant difference was observed across groups for the thermal pain threshold. The mechanical threshold for periorbital pain was only significantly reduced in rats administered Sample A. Immunoassays further indicated that serum levels of SP in the group administered Sample A were significantly higher than those in the control group; the serum levels of NO and CGRP were significantly higher in the group of rats receiving Sample B.

**Conclusion:**

We successfully developed an effective and safe rat model for investigating alcohol drink induced hangover headaches. This model could be used to investigate the mechanisms associated with hangover headaches for the development of novel and promising candidates for the future treatment or prophylaxis of hangover headaches.

## Introduction

Alcohol-induced headache (hangover headache) is among the most common types of headache, but the mechanism through which alcoholic drinks cause headaches remains unclear (1, 2). Most clinical studies of hangovers have been retrospective, and the pathophysiological mechanisms that lead to headaches caused by alcoholic drinks have not been well-studied. This research, focused on the pathogenesis of headaches associated with alcoholic hangovers, aims to facilitate the development of hangover headache therapies and could also improve the preparation process of alcoholic drinks to reduce the occurrence of hangover headaches (3, 4). However, few reports have described the development of animal models for the study of hangover headaches, and studies exploring the underlying mechanisms of hangover headaches are difficult to perform without an appropriate animal model.

A number of studies have shown that alcohol is an important headache trigger (5-9). Some studies have reported that alcohol-induced hangover headaches occur 4–24 hr after the end of alcohol intake and can cause migraine-like symptoms in individuals with a history of migraines, including unilateral throbbing pain and photophobia. Research has shown that although subjects with and without a history of headaches can experience hangover headaches, those with a headache history suffer from more severe forms of hangover headaches, even following the intake of a small amount of alcohol (10, 11). This finding indicates that subjects with a history of headaches may be more sensitive to headaches induced by alcoholic drinks. As summarized in previous reports, we believe that a persistent state that primes an individual for headache/migraine may be an important correlate of hangover headache incidence due to the occurrence of a neurogenic inflammatory response. To confirm our hypothesis, we developed a rat model of headache/migraine in a persistent state that was able to mimic the key features of headache. Based on the report by Oshinsky, the persistent headache state was induced by repeatedly stimulating the rat dura mater with an inflammatory soup (10). Headache exacerbation was triggered through the administration of varying doses of alcohol by oral gavage. Sample B (35.0% alcohol by volume) administered orally was able to successfully produce a rat model of hangover headaches.

The pathology of migraine/headache involves the sensitization and activation of trigeminal nociceptive neurons to promote hyperalgesia and allodynia (lowering of the pain threshold) (11, 12). A number of studies have confirmed that calcitonin gene-related peptide (CGRP) and substance P (SP), which are synthesized and released by primary afferent neurons, are key neuropeptide substances within the trigeminovascular system (TGVS). Persistent peripheral noxious stimulation increases CGRP and SP release, enhancing the transmission of nociceptive signals and inducing hyperalgesia (13-15). Nitric oxide (NO) is critical for headaches due to its role in the relaxation of cerebral arteries and pain transmission and the sensitivity of the central nervous system to NO (16). Some researchers have reported that serum NO levels significantly increase in rats experiencing headaches (17, 18). These data suggest that CGRP, SP, and NO are involved in the occurrence and development of headaches and might play crucial roles in headache pathophysiology. Therefore, we systematically evaluated headaches in rats using a range of behavioral and biological indicators, such as the response to mechanical and thermal pain and the concentrations of pain-related neuropeptides in the trigeminal nervous system, including serum levels of CGRP, SP, and NO. In this study, we aimed to generate and evaluate a rat model of hangover headaches. Analyzing various headache indices in the rat model could be used to investigate the mechanisms associated with hangover headaches for the development of novel and promising candidates for the future treatment or prophylaxis of hangover headaches. 

## Materials and Methods

***Animals***

Healthy Sprague Dawley (SD) rats (130 ± 20 g, specific pathogen-free, males) were obtained from the Experimental Animal Center of Chongqing Medical University (Certificate number: SCXK [YU] 2017-0001, Chongqing, China). Wistar rats (130 ± 20 g, specific pathogen-free, males) were obtained from the Liaoning Changsheng Biotechnology Company Ltd (Certificate number: SCXK [Liao] 2015-0001, Liaoning, China). All animals were acclimatized in the animal laboratory of the First Affiliated Hospital of Chongqing Medical University prior to experimentation. Rats were housed under optimal conditions for hygiene and temperature with 12-hr:12-hr dark:light photocycle and *ad libitum* access to food and water. The experimental plan for this study was approved by the Ethics Committee of the First Affiliated Hospital of Chongqing Medical University (20184801).

***Reagents***

The IS contained 1.0 mM bradykinin (BK), 1.0 mM histamine (HA), 1.0 mM 5-hydroxytryptamine (5-HT), and 0.1 mM prostaglandin E_2_ (PGE2) in phosphate-buffered saline (PBS, pH 7.4), purchased from Sigma (USA). Sample A (Trade name, Jingju, had been blended, and contained a variety of Traditional Chinese Medicines, including angelica, cistanche, and astragalus) was purchased from Jingbrand Co. Ltd (Hubei, China) and contained 35.16% alcohol by volume (Batch number: P1808121/06; Production date: 20180827). Sample B (Trade name, Jijiu) was also purchased from Jingbrand Co. Ltd and contained 35.0% alcohol by volume (Batch number: TJ18082501; Production date: 20180829). Sample C was normal saline (NS). The inflammatory soup was prepared as described previously(18-20). All prepared reagents were packaged individually and stored at −20 °C in the dark until use.

***Selection of animal strains and determination of the optimal dose of sample B-induced hangover***

All rats were trained for 3 days (once a day) for pole climbing, reversal reflex, and roller experiments before performing formal experiments. Training was performed in the morning (8:30 a.m.), and experimental rats that were unable to perform the pole climbing, reversal reflex, and roller experiments were eliminated from the study on the fourth day. SD rats who were successfully trained in the behavioral assessments were randomly divided into seven groups, based on the results of a preliminary experiment. Each group received a different AD sample dose. Rats were fasted for 12 hr prior to gavage, followed by the administration of different doses of Sample B by gavage: 0.02 l/kg (n = 10); 0.021 l/kg (n = 8); 0.022 l/kg (n = 10); 0.023 l/kg (n = 46); 0.024 l/kg (n = 12); 0.025 l/kg (n = 12); and 0.03 l/kg (n = 6). Experiments were performed in the morning (8:30 a.m.). We monitored the time points at which the righting reflex disappeared and recovered to determine the time required to become intoxicated and the total period of intoxication. The intoxicated proportion was calculated as the number of rats receiving AD samples that became intoxicated divided by the total number of rats receiving AD samples × 100%. The intoxicated death proportion was calculated as the number of rats that died after AD sample administration divided by the total number of rats receiving AD samples × 100%. The Wistar rats were then randomly divided into two groups, 0.023 l/kg (n = 10) and 0.025 l/kg (n = 10), based on the experimental results in SD rats for hangover onset, and the experimental results were recorded.

***Alcohol metabolism experiment***

Normal Wistar rats (n = 6) received an intragastrical (i.g.) administration of Sample B (ethyl alcohol, 35.0%, 0.025 l/kg), based on the results of the previous experiment. Blood was collected from the orbital vein at 19.5 hr, 22.0 hr, and 24.0 hr after i.g. administration of Sample B. Plasma was isolated, and the blood alcohol concentration (BAC) was measured by gas chromatography to determine the alcohol metabolism rate.

***Preparing the rat model of hangover headache***

**Figure 1:**
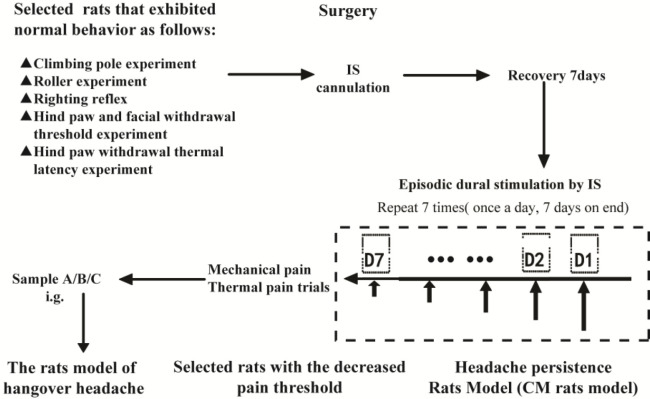
Schematic representation of the experimental design for Sprague Dawley rats

A schematic representation of the experimental design is shown in Figure 1. We selected Wistar rats that exhibited normal behaviors, as determined during the training period, to establish a chronic migraine (CM) rat model, followed by evaluating the outcomes of a pole climbing experiment, a roller experiment, righting reflex, hind paw withdrawal threshold, and hind paw withdrawal thermal latency. We used a surgical procedure to generate a rat model of CM, as described previously (10, 21). Rats were placed in a stereotaxic apparatus (ST-51063, Stoelting Co., Chicago, IL, USA), and deep anesthesia was induced with 10% chloral hydrate (0.4 g/kg body weight; intra-peritoneal injection). The bregma and midline bone sutures were identified, and craniotomy (1 mm in width) was performed above the superior sagittal sinus (SSS). A stainless-steel cannula (with a removable cap) was fixed to the bone around the opening in the skull using dental acrylic. This procedure allowed for direct access to the dura under sterile conditions. After surgery, 1.0 ml of procaine (5.0 mg/1.0 ml, once a day for 3 days) was applied under the surgical area of the skin to minimize the pain caused by the surgical incision. The rats were housed individually under optimal clean conditions, with controlled temperature and photoperiod (12 hr light:12 hr dark), and provided with food and water *ad libitum*. These rats were allowed to recover for at least 7 days before the inflammatory soup was applied to SSS. Seven days after recovery, we identified Wistar rats with no signs of infection that were in a good mental state (normal activity), and the hind paw withdrawal threshold was measured. Animals showing good levels of recovery during the hind paw withdrawal threshold experiment were selected to participate in subsequent experiments. Selected rats were randomly divided into a CM group (inflammatory soup) and a Sham group (PBS). Different groups of experimental rats were maintained in separate cages under the same conditions. Stimulation was performed by applying 3 µl of inflammatory soup or PBS to SSS through the dura cannula once per day for 7 days. CM rats that displayed a reduction in the hind paw withdrawal threshold were selected for inclusion in the AD-induced hangover headache experiments. The selected CM rats were randomly divided into three groups: CM+A group (CM + Sample A), CM+B group (CM + Sample B), and CM+c group (CM +Sample C). The three groups of rats received i.g. administrations of Samples A, B, or C at 0.025 l/kg. We measured the thresholds for the hind paw and face withdrawal and the hind paw withdrawal thermal latency. Detailed records were maintained after each gavage to determine the intoxication period, the intoxication latency period, and the proportion of intoxicated or dead rats in each experimental group.

***Distribution of hangover among chronic migraine rats***

The Sham and CM Wistar rats were randomly divided into Sample A groups (0.025 l/kg, n = 10) and Sample B groups (0.025 l/kg, n = 10), based on the results of previous experiments, and the experimental results were recorded.

***Evaluation of the rat model of hangover headache***

*Hind paw Withdrawal Threshold*

The hind paw withdrawal threshold was determined as previously described (10, 21). The experimental rats were maintained in a transparent cage measuring 22 cm × 22 cm × 30 cm for 30 min prior to the pain threshold measurement in a quiet environment. We used the electronic von Frey pain tester (2450 series: IITC Company of the United States, Woodland Hills, USA) to vertically stimulate the sole of the left paw on each rat. The pain threshold was determined as the reading displayed when the rat first lifted its leg. We measured the mechanical pain threshold in each rat prior to each dose of either inflammatory soup or physiological saline. Each test was repeated at least five times, with an interval of 2 min between tests. The pain threshold was determined as the mean value of three positive reactions.

*Facial withdrawal threshold*

The facial withdrawal threshold was measured according to the established methodology(22). Rats were placed in a fixator for 30 min and stimulated with 51000-20C Von Frey Hair Pain Test Kits (Danmic Company of the United States, USA). The test fibers were placed on the skin around the left and right eyes of the rats at different time points. We applied the fiber filaments for 1–2 sec, with intervals of 5 sec , and at least 30 sec intervals were used between different stimulation intensities. After stimulation, rats exhibited a range of reactions, including head retraction, scratching their faces with their front paws, and aggressive behavior. Any of these three manifestations was defined as a positive reaction. ‘No reaction’ was defined when no reaction occurred after fibers were bent at 90°, but stimulation with the same fiber elicited a positive reaction in more than 60% of cases (three of five total stimuli). The results were recorded as the mean value of the three intensities.

*Hind paw withdrawal thermal latency*

The measurement of hind paw withdrawal thermal latency utilized a radiant thermal pain meter. Room temperature was maintained at 25–30 °C during measurements. The rats were placed within the plexiglass frame of a pain-measuring instrument but were free to move at will. Once the rats became acclimatized to these surroundings, we applied a radiant heat source to the central region of the right hind paw and recorded the latencies of the hind paw withdrawal responses induced by thermal stimulation. The test was conducted three times, and the mean latency value was determined. The illumination interval was 5 min, the strength grade was 20%, and the upper time limit was 20 sec to prevent burning. The test was repeated five times, and the mean value of three positive reactions was used as the thermal pain threshold (23).

***Reversal reflex, pole climbing, and roller experiment***

Following AD gavage, the rats were placed gently into a box. If the rats maintained a posture with their backs facing downwards for more than 30 sec, then they were considered to be intoxicated (drunk). If the righting reflex recovered following intoxication, then the rats were considered to have recovered from intoxication (become sober)(23,24). Based on this behavior, rat intoxication can be accurately determined. The pole climbing and roller experiments were performed as described previously(25-28). Rats were trained for 2 days, once per day, before the formal experiment. The roller experiment and the pole climbing experiment were performed to investigate the recovery of physical strength in rats 24 hr after i.g. administration of AD Samples A or B. The rats were placed in a roller-coordinated motion detector (Shanghai Yuyan Biotechnology Co, Ltd, Shanghai, China, 30A), with an inclination of 45°, and rotating at a speed of 1.5 rpm/min. To maintain balance, rats that were not intoxicated and had recovered their physical strength would constantly adjust their bodies to avoid falling. The number of falls was recorded during a 5-min period. Pole climbing experiment: according to methods described by Ogawa *et al. *(28, 29), we constructed a 100-cm-long, 1-cm-diameter wooden pole and connected the bottom to a feeding cage filled with sawdust. Pole climbing training was performed for 2 days, once per day, prior to the formal experiment, and rats who failed to climb the pole were eliminated from the experiment. The rats were held by their tails, placed head down at the top of the pole, and allowed to climb down naturally. The time to climb down the pole was recorded and measured three times continuously to obtain a mean value. The failure to grasp the pole resulted in a time recorded as 0 sec.

***Detection of serum factors related to headache (CGRP, SP, and NO)***

Blood samples were collected from the periorbital venous plexus of experimental rats 24 hr after i.g. AD sample administration. Blood samples were then centrifuged at 3000 rpm/min for 10 min to separate the serum. Serum samples were subsequently used to measure the levels of CGRP, SP, and NO using specific enzyme-linked immunosorbent assay (ELISA) kits in accordance with the manufacturer’s instructions (Nanjing Jiancheng Biotechnology Co., Ltd., Jiangsu, China; NO, H217-1-2, H218-1-2, A012-1-2).

***Statistical analysis***

Data are expressed as the mean ± standard deviations or median and interquartile range. All data were compared using one-way ANOVA (non-parametric), and we use the Kruskal-Wallis test of the non-parametric test to analyze for not having a normal distribution, which has been corrected for multiple comparisons using statistical hypothesis testing by Tukey’s multiple comparisons test. For the normal distribution and heterogeneity of variance, we use Brown-Forsythe and Welch ANOVA tests to reanalyze those data, which have been corrected for multiple comparisons using statistical hypothesis testing by Dunnett’s T3 multiple comparisons test. All statistical analyses were performed using GraphPad Prism 8.0, and *P*<0.05 or *P*<0.01 were considered significant.

## Results

***Proportions of intoxication and death in Wistar and SD rats***

Table 1 shows the intoxication and mortality data following i.g. administration of Sample B (35.0% alcohol by volume) at doses of 0.02–0.030 l/kg. We found that the proportion of intoxicated SD rats was not positively correlated with sample dose; the proportions of intoxicated SD rats administered 0.023 l/kg, 0.025 l/kg, and 0.030 l/kg were 60%, 70%, and 66.7%, respectively, whereas the proportions of intoxicated rats that died were 4.3%, 8.3%, and 33.3%, respectively. Wistar rats were administered 0.023 l/kg and 0.025 l/kg by oral gavage, resulting in 70% and 90% intoxication, respectively. None of the Wistar rats died during these experiments.

***Hind paw withdrawal threshold and the hind paw withdrawal thermal latency at different time points***

**Figure 2:**
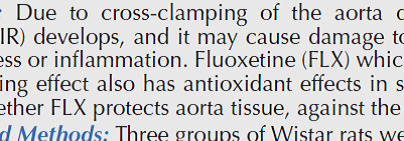
Hind paw withdrawal threshold and the hind paw withdrawal thermal latency at different time points. Sham: Phosphate-buffered saline (PBS, pH 7.4) stimulation was applied to the superior sagittal sinus (SSS) through a cannula in the dura that was affixed to the rat skull. CM: Episodic stimulation of the dura by applying 3 µl of IS to SSS (once per day for 7 days). Rats were tested at 0, 18, 21, 24, and 41 hr after Sample B administration. The black arrow indicates the minimum mechanical and thermal pain thresholds following i.g. administration of Sample B. N = 10 for each group; each graph shows the mean ± SD

PBS: Phosphate-buffered saline; CM: Chronic migraine; SSS: Superior sagittal sinus; NO: Nitric oxide; CGRP: Calcitonin gene-related peptide; SP: Substance P

**Figure 3:**
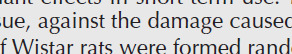
Hind paw withdrawal thresholds of rats in each group. Sham: PBS stimulation was applied to the superior sagittal sinus (SSS). CM: Episodic stimulation of the dura by applying 3 µl of IS to SSS (once per day for 7 days). CM+a: 24 hr after the CM group was administered sample A. CM+b: 24 hr after the CM group was administered Sample B. CM+c: 24 hr after the CM group was administered Sample C. a: JingJiu; Sample b: Jijiu; Sample c: normal saline, at a dose of 0.025 l/kg. The hind paw withdrawal threshold decreased significantly in the Sham groups compared with the CM group (*** *P*<0.001, *P<0.05, Sham group vs CM group); CM vs CM+a group, CM vs CM+c group; NS, *P*>0.05; CM vs CM+b group, *P<0.05 (n = 24, n = 15, and n = 9 rats in each group. The graph shows the median and interquartile range

PBS: Phosphate-buffered saline; CM: Chronic migraine; NS: Normal saline; SSS: Superior sagittal sinus

We measured the hind paw withdrawal threshold and the hind paw withdrawal thermal latency of rats in the Sham and CM groups at different time points after i.g. administration of Sample B (Figures 2A and B). Figure 3A shows that the hind paw withdrawal threshold of the CM group was lower than that of the Sham group. The hind paw withdrawal threshold gradually decreased after i.g. administration of Sample B, reaching a minimum of 18.39 g after 24 hr, which then increased to 24.88 g after 41 hr. Figure 2B shows that the hind paw withdrawal thermal latency of the CM group was lower than that of the Sham group at 0 hr after i.g. administration of Sample B. The thermal pain threshold of the CM group increased gradually, reaching a maximum of 20.64 g after 18 hr, but decreased to 13.25 g after 24 hr.

***Distribution of hangover onset after administration of alcohol drink samples to Wistar rats***

Administered Sample B at a dose of 0.025 l/kg, the proportion of Sham rats that became intoxicated in the group administered Sample A increased (95.2% *vs *82.9%), the time taken to become intoxicated was longer (51.8 min *vs* 49.73 min), and the total intoxication time was shorter (577 min* vs* 628 min). The proportion of rats that became intoxicated in the CM+A group decreased (51.85% *vs* 87.5%); the time to become intoxicated and the total intoxication time were also shorter (46.6 min *vs* 55.9 min, 450 min *vs* 555 min, respectively). Compared with the Sham group administered Sample B, the proportion of intoxicated rats in the CM+B group was higher (87.5% *vs* 82.9%), the time to become intoxicated was longer (55.9 min *vs* 49.73 min), and the total intoxication time was shorter (555 min* vs* 628 min). Compared with Sham rats administered Sample A, the proportion of intoxicated rates in the CM+A group was smaller (51.85% *vs* 95.2%), and the time to become intoxicated and the total intoxication time were shorter (46.6 min *vs *51.8 min, and 450 min *vs *577 min, respectively), all date were shown in Table 2.

***Hind paw withdrawal threshold***

The hind paw withdrawal threshold of each group is shown in Figure 3 A–C. The hind paw withdrawal threshold in our rat model of inflammatory soup-induced CM was significantly lower than that in the Sham group (*P*<0.001). The hind paw withdrawal thresholds in rats after 24 hr of intoxication in the CM+b group were significantly lower than in the CM group (*P*<0.05). No significant difference was observed between the CM+c and CM+a groups compared with the CM group.

***Hind paw withdrawal thermal latency***

**Figure 4:**
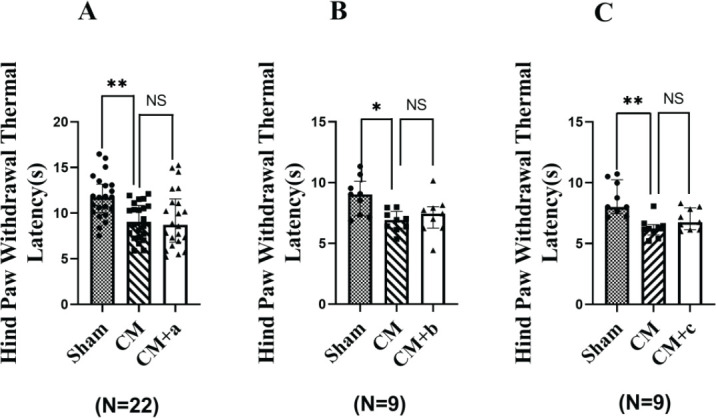
Hind paw withdrawal thermal latency in each group. Sham: PBS stimulation was applied to the superior sagittal sinus (SSS). CM: Episodic stimulation of the dura by applying 3 µl of IS to SSS (once per day for 7 days). CM+a: 24 hr after the CM group was administered sample A. CM+b: 24 hr after the CM group was administered Sample B. CM+c: 24 hr after the CM group was administered Sample C. a: Jing Jiu; Sample b: Jijiu; Sample c: normal saline, at a dose of 0.025 l/kg. The hind paw withdrawal thermal latency was significantly lower in the CM group; (** *P*<0.01, * *P*<0.05; Sham group vs CM group); NS (*P*>0.05), No significant differences between CM vs CM+a, CM+b, or CM+c groups. n = 22, n = 9, n = 9 rats in each group. The graph shows the median and interquartile range

PBS: Phosphate-buffered saline; CM: Chronic migraine; SSS: Superior sagittal sinus; NS: Normal saline

As shown in Figure 4A–C, the hind paw withdrawal thermal latency in the CM group was significantly lower (*P*<0.001, *P*<0.05) than that in the Sham group. There was no significant difference (*P*>0.05) between CM, CM+a group, CM+b group and the CM+c group in the thermal latency, 24 hr after i.g. administration of Samples A, B, and C.

***The facial withdrawal threshold***

**Figure 5:**
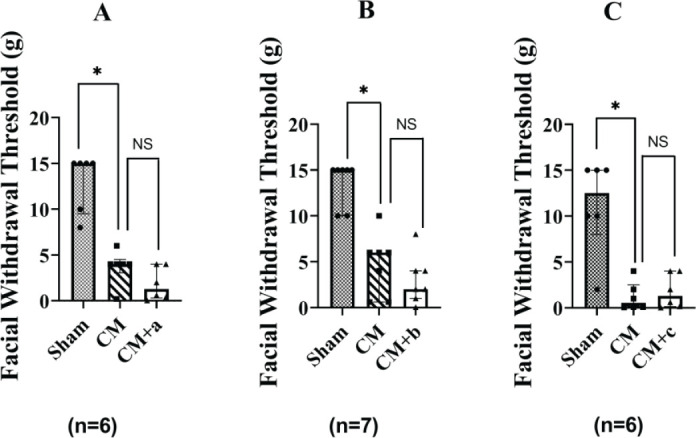
The facial withdrawal threshold of rats in each group. Sham: PBS stimulation was applied to the superior sagittal sinus (SSS). CM: Episodic stimulation of the dura by applying 3 µl of IS to SSS (once per day for 7 days). CM+a: 24 hr after the CM group was administered Sample A. CM+b: 24 hr after the CM group was administered Sample B. CM+c: 24 hr after the CM group was administered Sample c. a: Jingju; Sample b: Jijiu; Sample c: normal saline, at a dose of 0.025 L/kg. The mechanical pain threshold in the periorbital region was significantly lower in the CM group (Sham group vs CM group, *P<0.05), CM vs CM+a; NS (*P*>0.05), no significant differences between CM and CM+b or CM+c groups. n = 6, n = 7, n = 6 rats in each group. The graph shows the median and interquartile range

PBS: Phosphate-buffered saline; CM: Chronic migraine; NS: Normal saline; SSS: Superior sagittal sinus

As shown in Figure 5A–C, the facial withdrawal threshold in the Sham group was 5.0–10.0 g, whereas that of the CM group was significantly lower (* *P*<0.05). No significant differences (*P*>0.05) were observed between the CM, CM+a, CM+b, and CM+c groups.

***Analysis of serum factors associated with headache (SP, CGRP, and NO)***

**Figure 6:**
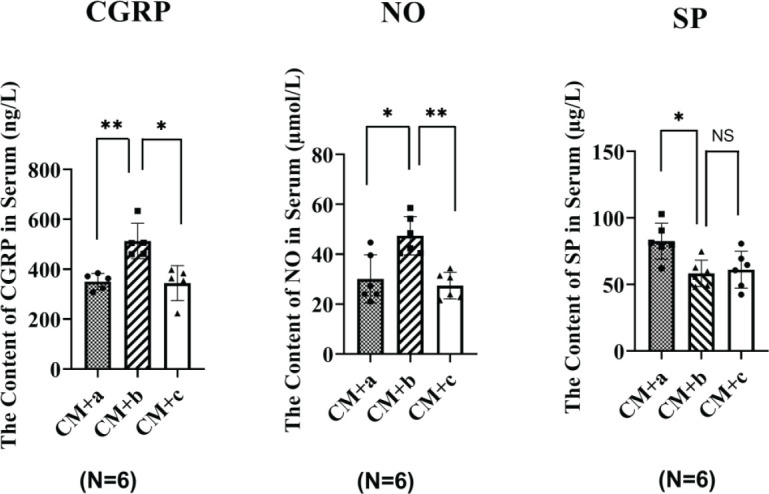
Serum levels of CGRP, NO, and SP in serum from different groups of rats. CM: Episodic stimulation of the dura by the application of 3 µl of IS to the superior sagittal sinus (SSS) (once per day for 7 days). CM+a: 24 hr after the CM group was administered Sample A. CM+b: 24 hr after the CM group was administered Sample B. CM+c: 24 hr after the CM group was administered Sample C. a: Jingju; Sample b: Jijiu; Sample C: normal saline, at a dose of 0.025 l/kg. The serum levels of CGRP were significantly higher in the CM+b group, (**P<0.01, CM+a vs CM+b; *P<0.05; **P<0.01, CM+b vs CM+c). The serum levels of NO were also significantly higher in the CM+b group (*P<0.05, CM+a vs CM+b; CM+b vs CM+c). The serum levels of SP were significantly higher in the CM+a group (*P<0.05, CM+a vs CM+b; CM+a vs CM+c; NS (P>0.05), with no significant differences between the CM+b and CM+c groups). N = 6 rats per group. The graph shows the mean ± SD

PBS: Phosphate-buffered saline; CM: Chronic migraine; NS: Normal saline; SSS: Superior sagittal sinus; NO: Nitric oxide; CGRP: Calcitonin gene-related peptide; SP: Substance P

The serum factors associated with headaches in experimental rats are shown in Figure 6. Analysis indicated that the CGRP levels in the serum from rats in the CM+b group were significantly higher than those in the CM+a and CM+c groups (*P*<0.01, *P*<0.05). The serum levels of NO in rats from the CM+a and the CM+c groups were significantly higher than those in the CM+b group (*P*<0.5, *P*<0.01). The serum levels of SP in the CM+b groups were significantly lower than those in the CM+a group (*P*<0.05), but no significant differences (*P*>0.05) were observed between CM+b and CM+c groups.

***Analysis of the degree of headache observed in experimental rats***

**Figure 7:**
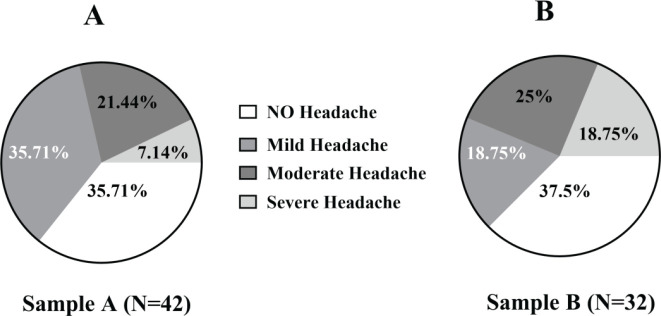
A schematic diagram showing different grades of headache following alcohol intoxication. Sample A (n = 42) is Jing Jiu, containing 35.0% alcohol by volume. Sample B is Jijiu (n = 32) which contained 35.16% alcohol by volume. After drinking Sample A or B, rats with hangover headaches were divided into four categories according to the degree of reduction in the hind paw withdrawal threshold: no headache (hind paw withdrawal threshold showed no change after alcohol administration); mild headache (a reduction in the hind paw withdrawal threshold of 0%–30% after alcohol administration); moderate headache (a reduction in the hind paw withdrawal threshold of 30%–50% after alcohol administration); and severe headache (a reduction in the hind paw withdrawal threshold of over 50% after alcohol administration). The degree of reduction in the hind paw withdrawal threshold was determined as follows: (hind paw withdrawal threshold before drinking Sample A or B − hind paw withdrawal threshold after drinking Sample A or B) / hind paw withdrawal threshold before drinking Sample A or B × 100%. Analysis showed that 64.8% of the rats administered Sample A experienced hangover headaches, with mostly mild (35.71%) or moderate (21.44%) levels. By contrast, 62.5% of rats administered Sample B experienced hangover headaches, which were mostly moderate (18.75%) or severe (25%)

According to Figures 7A and B, 64.8% and 62.5% of CM rats suffered from headaches after i.g. administration of Sample A and Sample B, respectively. However, 35.72% of rats in the CM group receiving Sample A did not suffer from headaches, compared with 37.5% of rats in the CM group receiving Sample B. The headaches experienced by the group receiving Sample A were primarily classified as mild or moderate (35.71% and 21.43%, respectively). By contrast, the rats receiving Sample B experienced headaches that were classified as either moderate or severe (18.75% and 25%, respectively). Only 7.14% of rats receiving Sample A developed severe headaches.

**Table 1:** Proportions of intoxicated and intoxicated-induced death among Wistar and SD rats following administration of various doses of Sample B

Dosage （L/kg）of Sample B*	SD (%)		Wistar(%)	
	Proportions of intoxication	Proportions of intoxicated that death	Proportions of intoxication	Proportions of intoxicated that death
0.020	10 (N=10)	0	\	\
0.021	12 (N= 8)	0	\	\
0.022	40 (N=10)	0	\	\
0.023	60 (N=46)	4.3	70 (N=10)	0
0.024	40 (N=12)	0	\	\
0.025	70 (N=12)	8.3	90 (N=10)	0
0.030	66.7 (N=6)	33.3	\	\

*Sample B (Jijiu, alcohol content: 35.0% by volume). N shows the number of rats in each experimental group

**Table 2:** Distribution of onset of hangover after administration of alcohol drinks samples to Wistar rats

Grouping	Number of rats	Proportions of intoxication (%)	Latent period of intoxication (min)	Period of sobered up (min)
a	21	95.2	51.8	577
b	41	82.9	49.73	628
CM+ a	27	51.85	46.6	450
CM+ b	16	87.5	55.9	555
CM+ c	9	0	-	-

a: Sample A, JingJiu; b:Sample B, Jijiu; c:Sample C, normal saline, at a dose of 0.025 L/kg.

## Discussion

A previous paper by Oshinsky reported that recurrent episodes of headache could be simulated by the repeated stimulation of the dura mater in rats using an IS to establish a CM rat model, in which rats experience a long-term persistent headache state(10,30-32). We used this model to investigate headaches induced by alcoholic drinks. First, we selected rats that displayed normal behaviors based on the results of pole climbing, roller experiment, righting reflex, mechanical pain, and thermal pain evaluations. Second, we established a rat model of CM through administration of IS. Third, we triggered headaches through i.g. administration of AD samples to obtain a stable and repeatable rat model of hangover headache. We systematically evaluated a range of behavioral indicators, including the hind paw withdrawal threshold and the hind paw withdrawal thermal latency, and measured pain-associated factors in the serum, including CGRP, SP, and NO. Thus, we generated a systematic model with which to evaluate the effects of AD on headaches through evaluation of behavioral and pain factors (Figure 1).

In this study, we used Wistar and SD rats in an intoxication experiment. Our data showed that Wistar rats consistently became intoxicated after i.g. administration of Sample B (35.0% alcohol by volume) at doses of 0.020– 0.030 ml/kg body weight. Table 1 shows that stable, high proportions of intoxication and low proportions of death were observed in Wistar rats compared with SD rats. These differences are likely due to variations between strains; thus, we chose Wistar rats as the experimental model for our hangover headache experiments. Preliminary experiments revealed that the best dose of Sample B was 0.025 ml/kg body weight in Wistar rats, which was the dose used for further experiments.

We used inflammatory soup to stimulate SSS area of the dura mater for 7 days to create a rat model of persistent headache, CM. Then, we measured the hind paw withdrawal threshold and the hind paw withdrawal thermal latency at various time points after i.g. administration of Sample B. The results have demonstrated that the hind paw withdrawal thermal responses increased after the administration of Sample B, which may be related to insensitivity to thermal pain following alcohol intoxication, consistent with the results shown in Figure 2. In the results of hind paw withdrawal thermal latency and facial withdrawal threshold, CM+a, CM+b, and CM+c groups, there were no significant differences (*P*>0.05) observed between CM and CM+a, CM+b and CM+c groups (Figures 3 and 4). Therefore, we did not use hind paw withdrawal thermal latency and facial withdrawal threshold to evaluate headache after an alcohol hangover. The hind paw withdrawal threshold decreased significantly in the CM+b groups compared with the CM group, and no significant differences (*P*>0.05) were observed between CM and CM+a. The reason may be that some alcoholic drinks containing traditional Chinese medicine ingredients might provide protective effects against hangover headaches. The hind paw withdrawal threshold reduced after AD administration, consistent with previous research (33-36). The alcohol metabolism experiment showed that 25% of rats completely metabolized the alcohol in their blood within 19.5 hr after administration. After 22 hr, 75% of the rats had completely metabolized the alcohol in their blood. By 24 hr, all of the rats had completely metabolized the alcohol in their blood. The hind paw withdrawal threshold was lowest 24 hr after AD administration (18.39 g). The pole climbing and roller experiments showed that the rats displayed normal behavior 23 hr after intoxication, indicating that the rats were no longer intoxicated and their physical strength had recovered. A hangover headache is clinically defined as a headache that causes migraine-like symptoms 4–24 hr after alcohol intake (36). Therefore, we measured the hind paw withdrawal threshold in rats with hangover headaches 24 hr after AD administration. Sample B consists of an unblended wine containing some alcohols, higher alcohols, fuseo alcohols, aldehyde compounds, and so on. Nearly all white wines and healthcare liquor are blended from this type of wine in the liquor industry. Although any alcohol can be used to prepare a hangover model, unblended wine (ji jiu) was used to prepare our hangover headache rat model using oral gavage to simulate clinic practice in this paper.

The data in Figure 3 show that the hind paw withdrawal threshold significantly decreased in the CM+A and CM+B groups compared with the CM group. The proportion of intoxicated rats in the B group was lower than that in the CM+B group (82.9% *vs *87.5%). The time to intoxication was shorter in the B group than in the CM+B group (49.7 min *vs* 55.9 min), as shown in Table 2. The lower nociceptive threshold in sensitized rats with a history of repeated inflammatory soup-mediated trigeminal nociceptor stimulation may represent a key pathological foundation for understanding hangover headaches. These results confirm our hypothesis that subjects who have a history of headaches may be more sensitive to headaches induced by alcohol. Alcoholic drinks induce short headaches in non-migraineurs, whereas alcoholic drinks in migraine patients can trigger a long-lasting migraine-like hangover headache. These data indicated that this CM rat model, generated by central and peripheral sensitization, experienced a more severe AD-induced hangover headache. Previous authors have speculated that recurrent migraines can cause changes in the neural circuitry, resulting in increased neuronal excitability and increased susceptibility to hangover headaches (37). Consequently, a rat model of peripheral or central sensitization generated by the repeated stimulation of SSS using inflammatory soup can better simulate the occurrence of hangover headache following i.g. administration of AD than rats that have not been pre-stimulated.

Previous studies have reported that serum levels of CGRP, NO, and SP are associated with pain, and increased serum levels of these factors are positively related to the level of pain experienced (13,18,15). CGRP, as a key neuropeptide in the trigeminal system, has been implicated in the peripheral and central sensitization of headache(37), and CGRP levels are increased during headache events in patient serum and cerebrospinal fluid and the serum of animal models of headache (38). Figure 6 shows that the serum levels of SP in rats with hangover headache following i.g. administration of Sample A were significantly higher than those in rats administered Sample B or C, but no significant difference was observed between administration of Samples B and C (*P*>0.05). The serum levels of CGRP and NO in rats with hangover headaches following Sample B administration were significantly higher than those in rats administered Samples A or C (*P*<0.05). These results are inconsistent with previous reports, which may be due to the presence of traditional Chinese medicine ingredients in Sample A, including angelica, cistanche, and astragalus, which might inhibit CGRP and NO generation, although the specific mechanisms remain unclear. Moreover, some components in alcoholic drinks might also upregulate inflammatory factors (IL-1, IL-6, and TNF-α), causing neurogenic inflammation and peripheral sensitization, increasing plasma levels of CGRP and NO. By contrast, the complex components in alcoholic drinks might inhibit SP levels in the plasma. To avoid potential interference, we did not use SP to evaluate the occurrence of hangover headaches. According to Figure 7A and B, the proportion of CM rats that developed a headache following the administration of Samples A and B were 64.8% and 62.5%, respectively. The types of headaches experienced by CM rats following Sample A administration were primarily mild or moderate. By contrast, the types of headaches experienced by CM rats following administration of Sample B were primarily moderate and severe (18.75% and 25%, respectively). The incidence of severe headaches in the CM+A group was only 7.14%. Compared with the CM+C group, the serum levels of SP in rats receiving Sample A were higher, which may be associated with the development of headache in 64.8% of CM rats following administration of Sample A, and the data were shown in Figure 6. Therefore, the addition of traditional Chinese medicine components to Sample A to inhibit SP levels may reduce the incidence of headache following intoxication. Furthermore, Table 2 shows that the proportion of intoxicated rats in the CM+B was higher than in the Sample B group (87.5% *vs *82.9%), which is consistent with clinical studies reporting increased hangover vulnerability among patients with a history of recurrent headaches (35, 38, 39). We repeatedly administered IS topically onto SSS of the dura mater to activate the trigeminal nerve system and induce neurogenic inflammation and CGRP release, which appeared to increase the incidence of recurrent headaches. Neurogenic inflammation may increase Ca^2+^ permeability, leading to toxic Ca^2+^ influx. When Ca^2+^ reaches a critical concentration in neurons, calmodulin binds to Ca^2+^ to activate NOS, leading to a significant increase in NO synthesis, which may induce central hyperalgesia and aggravate headache attacks (18, 20). Neurogenic inflammation (by inflammatory soup or alcohol) causes the dura, trigeminal ganglion, and trigeminal nucleus caudalis to release CGRP into the blood. Hangover severity in patients with a history of headaches may be associated with increased CGRP and NO release, aggravating the inflammatory response. Some researchers have indicated that biomarkers of alcohol metabolism, oxidative stress, and the inflammatory response may represent potentially important determinants of hangover severity (40). The proportion of intoxicated rats in the CM+A group was lower than that in the CM+B group (51.85% *vs *87.5%), and the time to intoxication and the total time of intoxication were both shorter for the CM + A group than the CM + B group (46.6 min *vs* 55.9 min, 450 min *vs* 555 min). Therefore, our data suggest that the presence of traditional Chinese medicine ingredients in Sample A may provide protective effects against the development of headaches after i.g. administration of ADs.

## Conclusion

We successfully generated a rat model of CM through central and peripheral sensitization by repeatedly stimulating SSS of the dura mater in Wistar rats with IS. I.g. administration of Sample B successfully stimulated headache onset. This model was effective and stable and could be used to investigate hangover headaches. Furthermore, we demonstrated that the hind paw withdrawal threshold and serum levels of NO and CGRP could be used to quantitatively evaluate the degree of a hangover headache. Alcoholic drinks containing traditional Chinese medicine ingredients might provide protective effects against hangover headaches.

## Authors’ Contributions

SL, YZ, and YY wrote the manuscript; Yafang Z, GQ, QF, YS, FZ, and ZW performed data analysis; YC, YL, and LC designed the study. All authors read and approved the final version of the manuscript.

## Conflicts of Interest

There are no conflicts of interest.
